# RNA splicing modulator for Huntington’s disease treatment induces peripheral neuropathy

**DOI:** 10.1016/j.isci.2025.112380

**Published:** 2025-04-08

**Authors:** Florian Krach, Tom Boerstler, Stephanie Neubert, Laura Krumm, Martin Regensburger, Jürgen Winkler, Beate Winner

**Affiliations:** 1 Department of Stem Cell Biology, University Hospital Erlangen, Friedrich-Alexander Universität Erlangen-Nürnberg (FAU), Kussmaulallee 4, 91054 Erlangen, Germany; 2 Department of Molecular Neurology, University Hospital Erlangen, Friedrich-Alexander Universität Erlangen-Nürnberg (FAU), Schwabachanlage 6, 91054 Erlangen, Germany; 3 Center for Rare Disorders (ZSEER), University Hospital Erlangen, Friedrich-Alexander Universität Erlangen-Nürnberg (FAU), Kussmaulallee 4, 91054 Erlangen, Germany; 4 Deutsches Zentrum Immuntherapie (DZI), University Hospital Erlangen, Friedrich-Alexander Universität Erlangen-Nürnberg (FAU), Ulmenweg 18, 91054 Erlangen, Germany

**Keywords:** Pharmacology, Natural sciences, Biological sciences, Neuroscience, Cellular neuroscience, Cell biology

## Abstract

RNA splicing modulators, a new class of small molecules with the potential to modify the protein expression levels, have quickly been translated into clinical trials. These compounds hold promise for treating neurodegenerative disorders, including branaplam for lowering huntingtin levels in Huntington’s disease. However, the VIBRANT-HD trial was terminated due to the emergence of peripheral neuropathy. Here, we describe the complex mechanism whereby branaplam activates p53, induces nucleolar stress in human induced pluripotent stem cell (iPSC)-derived motor neurons (iPSC-MN), and thereby enhanced expression of the neurotoxic p53-target gene BBC3. On the cellular level, branaplam disrupts neurite integrity, reflected by elevated neurofilament light chain levels. These findings illustrate the complex pharmacology of RNA splicing modulators with a small therapeutic window between lowering huntingtin levels and the clinically relevant off-target effect of neuropathy. Comprehensive toxicological screening in human stem cell models can complement pre-clinical testing before advancing RNA-targeting drugs to clinical trials.

## Highlights

• Multi-omic analysis predict branaplam-induced p53 activation
• Branaplam alters rRNA processing and leads to nucleolar stress
• Branaplam leads to expression of neurotoxic p53-target BBC3
• Treated iPSC-MN show increase neurite disintegration with elevated NfL release

## Graphical abstract

**Figure:**
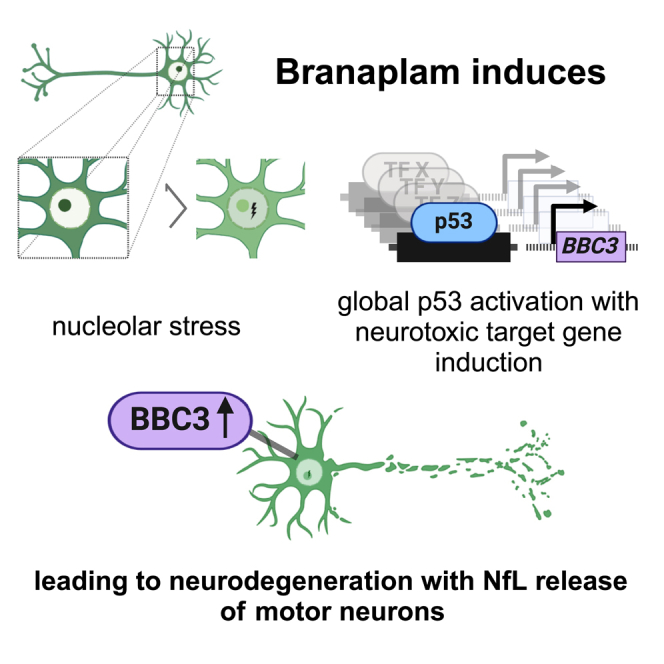

## Introduction

Branaplam is a small molecule RNA splicing modulator promoting changes in alternative splicing of the survival of motor neuron 2 (SMN2) mRNA^1^ and underwent clinical investigation in spinal muscular atrophy (SMA) (NCT02268552). One of branaplam’s off-target RNAs opened up an exciting chance to treat Huntington’s disease (HD). HD is a neurodegenerative disorder linked to CAG-repeat expansion in the huntingtin (HTT) gene, resulting in a toxic poly-Q protein. We and others have shown that branaplam induces a frame-shift-inducing pseudo-exon in the HTT transcript that destabilizes the HTT RNA and reduces mutant HTT protein expression.^2^^,^^3^ Hence, a phase II clinical trial was initiated to treat HD (NCT05111249). The trial was suspended in 2022. In a community update to the HD community by the sponsor, an increase in serum neurofilament light chain (NfL) and neurological symptoms and nerve conduction studies consistent with peripheral neuropathy were reported in branaplam-treated individuals.^4^ However, it remains elusive how the RNA splicing modulator leads to peripheral neuropathy. To solve this issue, human-based models can aid in identifying adverse events before initiating a clinical study.

Here, we describe the cellular mechanism of how the branaplam leads to peripheral neuropathy and NfL increase. Human iPSC-derived motor neurons treated with branaplam exhibit neurite fragmentation and increased NfL in the medium. We re-analyze RNA sequencing (RNA-seq) datasets of branaplam-treated human fibroblasts and identify a clear signal of p53-target gene activation. We evaluate the origin of p53 activation and reveal increased nucleolar stress upon branaplam in human iPSC-derived motor neurons. Branaplam-treated neurons induce expression of the neurotoxic p53-target BCL2 binding component 3 (BBC3).

## Results

### Branaplam leads to axonal degeneration and NfL increase

**Figure 1:**
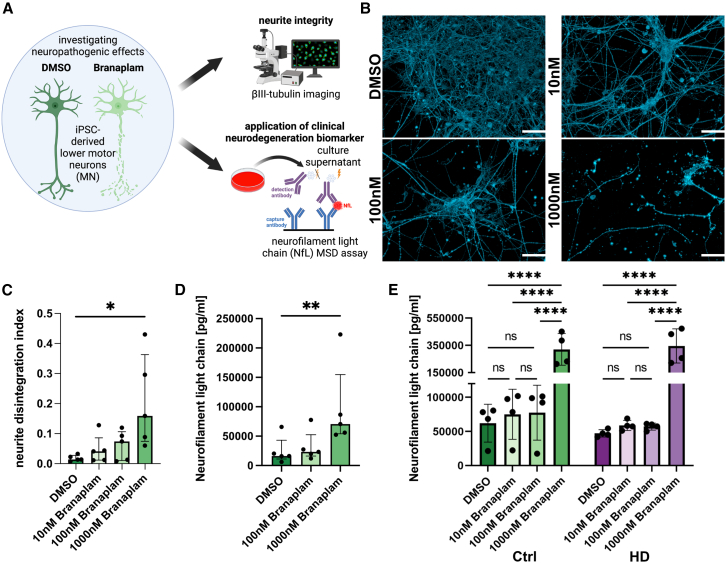
Branaplam leads to axonal degeneration and NfL increase in human iPSC-MN (A) Paradigm illustrating experimental approach. (B) Representative pictures of beta-III-Tubulin in DMSO and 1,000 nM-treated conditions. All shown images are from UKERiff2-X-18. Scale bar: 50 μm. (C) Bar plot showing quantification of neurite disintegration index (= disintegrated area (betaIII-Tubulin intensity above mean + 3× standard deviation of DMSO)/total area (beta-III-Tubulin area). Dots depict individual values per cell line (*n* = 5), graph as median with interquartile range. Statistics: Friedman test: *p* value: 0.0167. Dunn’s multiple comparison test: ∗ < 0.05; ∗∗ < 0.01; ∗∗∗ < 0.001. Only 1,000 nM passed the significance threshold. (D) Bar plot showing quantification of neurofilament light chain (NfL) levels in media supernatant of iPSC-derived neurons after 5 days of treatment with DMSO, 100 nM, or 1,000 nM branaplam. Dots depict individual values per cell line (n = 5), graph as median with interquartile range. Statistics: Friedman test: *p* value: 0.0008. Dunn’s multiple comparison test: ∗ < 0.05; ∗∗ < 0.01; ∗∗∗ < 0.001. Only 1,000 nM passed the significance threshold. (E) Bar plot quantification of neurofilament light chain (NfL) levels in media supernatant of iPSC-derived neurons in Ctrl (greens, *n* = 4) and HD (purples, *n* = 4) treated for 5 days with 10 nM, 100 nM, or 1,000 nM branaplam (opaques). Dots depict individual values, graph as median with interquartile range. Statistics: paired two-way ANOVA: *p* value (treatment): < 0.0001; *p* value (disease): 0.8352; *p* value (interaction): 0.5045. Šídák’s multiple comparisons test: ∗ < 0.05; ∗∗ < 0.01; ∗∗∗ < 0.001.

Peripheral neuropathy affects neurons’ axons that are extended outside the central nervous system, e.g., lower motor neurons. To test whether cellular signs of peripheral axonopathy induced by branaplam can be measured *in vitro*, we differentiated induced pluripotent stem cells (iPSCs) from healthy individuals into lower motor neurons (iPSC-MN) using a previously established protocol^5^ and investigated the toxic effect on human neurites (Figure 1A). In iPSC-MN treated for 5 days with branaplam, we detected more disintegrated neurites, consistent with axonal degeneration observed in neuropathies (Figures 1B and 1C). Strikingly, we detected increased levels of NfL in the supernatant of branaplam-treated iPSC-MN (Figure 1D). We next asked if there is a potential disease-drug interaction. To evaluate potential exacerbation in HD patients, we quantified NfL levels in Ctrl and HD-derived iPSC-MN using different doses of branaplam treatment. While branaplam leads to an NfL increase at higher doses in Ctrl and HD, there is no statistically significant indication of an interaction between branaplam treatment and HD (Figure 1E). This illustrates that our cellular model recapitulates cytopathogenic features coherent with the clinically observed parameters of neuropathy and elevated NfL in the phase 2 HD clinical trial.

To uncover a direct mRNA-mediated mechanism, we leveraged our human fibroblast and iPSC-neuron RNA-seq dataset of Ctrl and HD patients treated with the low dose of 10 nM branaplam for 72 h resulting in a significant reduction of mHTT protein levels^3^ (Figure S1A). We investigated if branaplam targets genes known to cause neuropathy in a monogenic manner by comparing our results with two independent genetic panels (Figure S1B). We found that one branaplam target appears also in a neuropathy gene panel (Figure S1C). Branaplam induces an in-frame exon inclusion in the protein O-mannosyltransferase 2 (POMT2) (Figure S1D). The insertion does not lead to a frameshift or insertion of an in-frame premature STOP codon. While the functional consequence of this exon inclusion is unclear, POMT2 is linked to muscular dystrophies, i.e., dystroglycanopathies.^6^^,^^7^ However, neuropathic features have not been described to our knowledge, making it an unlikely culprit. We further examined whether branaplam treatment indirectly affects the expression of neuropathy-associated genes, but we did not observe substantial effects (Figures S1E–S1H).

### Branaplam leads to p53 activation and cell-cycle arrest

**Figure 2:**
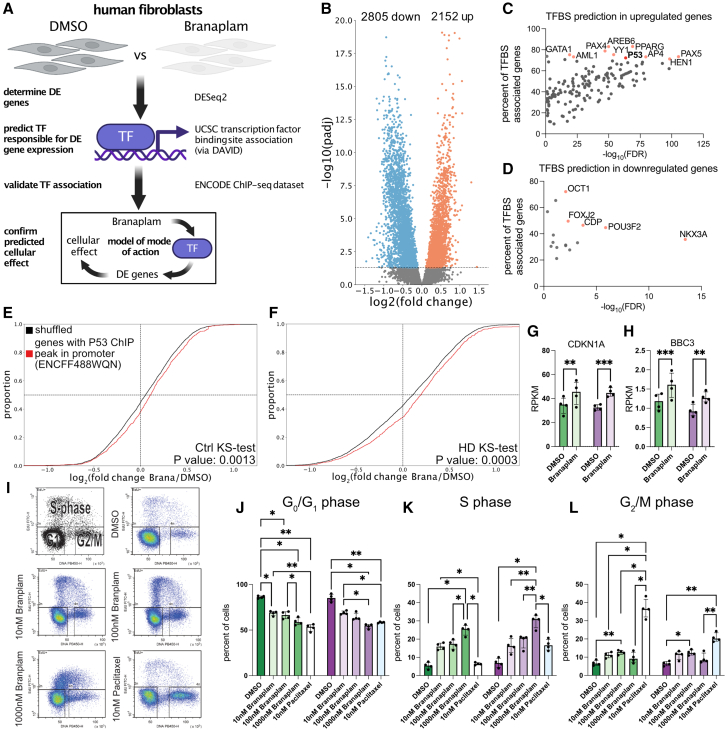
Branaplam leads to p53 activation and cell-cycle arrest (A) Experimental analysis strategy. DMSO (*n* = 8) vs. branaplam (*n* = 8) treated fibroblast RNA-seq data^3^ was used to determine differential gene expression and analysis of enrichment of transcription factor (TF) binding sites (TFBS), followed by validation using ENCODE ChIP-seq data and determine of the cellular mode of action. (B) Volcano plot of differentially expressed genes (blue: downregulated, red: upregulated). y axis depicts negative log_10_ of the adjusted *p* value from the DESeq2 output. (C) Scatterplot of significantly enriched (x axis, false discovery rate [FDR]) TFBS in genes upregulated upon branaplam treatment. y axis depicts percentage genes associated TF that are differentially upregulated upon branaplam. In red are 10 TFs with high significance and percentages. (D) Scatterplot of significantly enriched (x axis, false discovery rate [FDR]) TFBS in genes downregulated upon branaplam treatment. y axis depicts percentage genes associated TF that are differentially downregulated upon branaplam. In red are 5 TFs with high significance and percentages. (E) Cumulative distribution plot of log_2_(fold changes) of branaplam vs. DMSO in Ctrls in genes with p53 ChIP-seq peak within region of 5,000 bp upstream of gene start (red, *n* = 865 genes) or in a randomly shuffled background of equal size (black, *n* = 865 genes). Significance calculated with 2 sample Kolmogorov-Smirnov (KS) test. (F) Cumulative distribution plot of log_2_(fold changes) of branaplam vs. DMSO in HD in genes with p53 ChIP-seq peak within region of 5,000 bp upstream of gene start (red, *n* = 865 genes) or in a randomly shuffled background of equal size (black, *n* = 865 genes). Significance calculated with 2 sample Kolmogorov-Smirnov (KS) test. (G) Bar plot of CDKN1A RPKM values with Ctrl (greens, *n* = 4) and HD (purples, *n* = 4) fibroblast with DMSO and branaplam (opaque) treatment. Dots depict individual values, graph as median with interquartile range. Statistics: paired two-way ANOVA: *p* value (treatment): < 0.0001; *p* value (disease): 0.8851; *p* value (interaction): 0.3535. Šídák’s multiple comparisons test: *p* value (Ctrl): 0.001; *p* value (HD): 0.0004. (H) Bar plot of BBC3 RPKM values with Ctrl (greens, *n* = 4) and HD (purples, *n* = 4) fibroblast with DMSO and branaplam (opaque) treatment. Dots depict individual values, graph as median with interquartile range. Statistics: paired two-way ANOVA: *p* value (treatment): <0.0001; *p* value (disease): 0.1239; *p* value (interaction): 0.3071. Šídák’s multiple comparisons test: *p* value (Ctrl): 0.0006; *p* value (HD): 0.002. (I) Paradigm of EdU FACS cell cycle analysis and example scatterplots and gatings from DMSO, 10 nM branaplam, 1,000 nM branaplam and paclitaxel of UKERf33Q. (J) Bar plot of percentages of cells in G_0_/G_1_ phase in Ctrl (greens, *n* = 4) and HD (purples, *n* = 4) treated for 72h with 10 nM, 100 nM, 1,000 nM branaplam (opaques) or 24 h 10 nM paclitaxel (white/blue). Dots depict individual values, graph as median with interquartile range. Statistics: paired two-way ANOVA: *p* value (treatment): < 0.0001; *p* value (disease): 0.7840; *p* value (interaction): 0.0166. Šídák’s multiple comparisons test: ∗ < 0.05; ∗∗ < 0.01; ∗∗∗ < 0.001. (K) Bar plot of percentages of cells in S phase in Ctrl (greens, *n* = 4) and HD (purples, *n* = 4) treated for 72 h with 10 nM, 100 nM, 1,000 nM branaplam (opaques) or 24 h 10 nM paclitaxel (white/blue). Dots depict individual values, graph as median with interquartile range. Statistics: paired two-way ANOVA: *p* value (treatment): < 0.0001; *p* value (disease): 0.0345; *p* value (interaction): 0.0018. Šídák’s multiple comparisons test: ∗ < 0.05; ∗∗ < 0.01; ∗∗∗ < 0.001. (L) Bar plot of percentages of cells in G_2_/M phase in Ctrl (greens, *n* = 4) and HD (purples, *n* = 4) treated for 72h with 10 nM, 100 nM, 1,000 nM branaplam (opaques) or 24 h 10 nM paclitaxel (white/blue). Dots depict individual values, graph as median with interquartile range. Statistics: paired two-way ANOVA: *p* value (treatment): < 0.0001; *p* value (disease): 0.0127; *p* value (interaction): <0.0001. Šídák’s multiple comparisons test: ∗ < 0.05; ∗∗ < 0.01; ∗∗∗ < 0.001.

We reasoned that branaplam may mediate toxic neuropathic features via mechanisms other than targeting mRNA. Hence, we studied the cellular state of branaplam-treated fibroblasts by delineating the pathways altered upon treatment. Specifically, we investigated the transcription factor (TF) binding sites (TFBS) of differentially expressed genes (Figures 2A–2D). Genes that are downregulated upon branaplam treatment did not show a large number of significant enrichments of TFBS (Figure 2D). However, genes that are upregulated upon branaplam exhibited a large number of highly significant TFBS (Figure 2C). Strikingly, the tumor suppressor gene p53 was among the top 10 TFs with the highest percentage of shared genes. Inhibition of p53 results in amelioration of chemotherapy-induced neuropathy.^8^^,^^9^^,^^10^^,^^11^ Furthermore, p53 activation and upregulation of its downstream target BBC3 (in mice known as Puma) have been extensively studied as major inducers of axonal degeneration.^12^^,^^13^^,^^14^ Next to p53, literature research revealed PPARG and YY1 as potentially relevant. Inhibitors of PPARG ameliorated phenotypes in mouse models of chemotherapy-induced and diabetic neuropathy due to its neuroprotective properties,^15^^,^^16^ but a direct link connecting PPARG to peripheral neuropathy is not evident. Changes in expression levels of YY1 are associated with different forms of peripheral neuropathy. YY1 expression was reduced in critical illness neuropathy^17^ and upregulated in paclitaxel-induced neuropathy.^18^ However, in branaplam-treated cells, YY1 expression does not change (log_2_FoldChange: 0.096; adj. *p* value: 0.151). Hence, we concluded that PPARG and YY1 may not be the main culprits in this case. To our knowledge, no other transcription factors are known to be associated with peripheral neuropathy. Therefore, we moved further on with investigations on p53. We integrated an ENCODE p53 chromatin immunoprecipitation sequencing (ChIP-seq) dataset^19^^,^^20^ and analyzed gene expression changes upon branaplam treatment in Ctrl and HD fibroblasts in genes with a p53 ChIP-seq peak in a location of up to 5,000 bp upstream of the gene start. Strikingly, significantly higher gene expression was observed in Ctrl and HD fibroblasts upon branaplam treatment of p53 targets compared to a shuffled background (Figures 2E and 2F). Besides the well-known transcriptional activation of CDKN1A (p21), p53-mediated gene activation is also known to upregulate BBC3. Interestingly, both CDKN1A and BBC3 were upregulated upon branaplam treatment in fibroblasts (Figures 2G and 2H). Together, these data suggest a global activation of p53 signaling. p53’s function as a tumor suppressor to attenuate proliferation is widely known. Hence, we predicted that fibroblasts treated with branaplam show an altered cell cycle. Since 10 nM branaplam did not yield proliferation changes in cortical progenitors,^3^ we used two higher doses (100 nM and 1000 nM, 72 h treatment) (Figure 2I). To probe for shared mechanisms, we treated with paclitaxel, a cytostatic agent leading to an M-phase arrest, clinically used for the cancer treatment. A frequent and clinically relevant adverse reaction of paclitaxel is peripheral neuropathy. This adverse drug reaction occurs in more than one out of ten individuals across various formulations according to data available in the summary of product characteristics and prescribing information from market access authorization (e.g., European Medicines Agency, Abraxane^21^). As expected, paclitaxel leads to an M-phase arrest. Branaplam also alters the cell cycle in a dose-dependent manner but leads to a G_1_/S-phase arrest. As expected from our NfL measurements (Figure 1E), we did not observe a disease-drug interaction in this experiment (Figures 2I–2L). Together, this suggests that branaplam treatment induced cell-cycle arrest via p53 activation but in a non-paclitaxel-like manner (Figures 2I–2L).

### Branaplam leads to nucleolar stress

**Figure 3:**
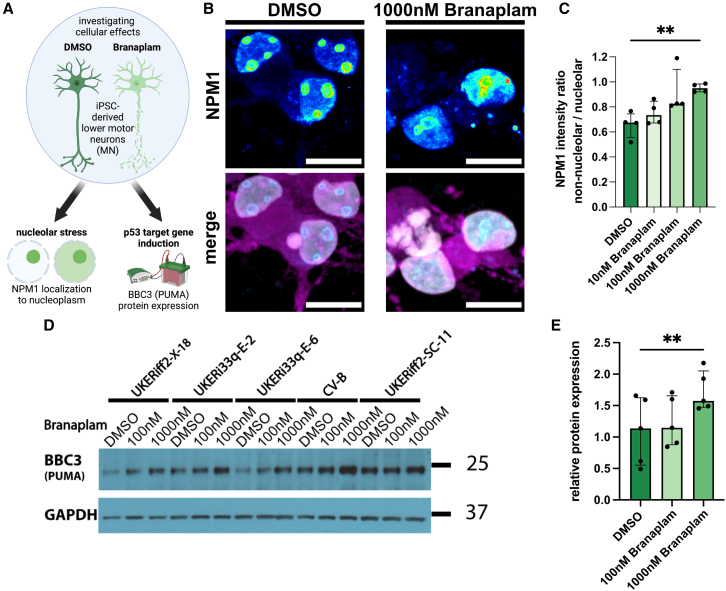
Branaplam leads to activation of nucleolar stress and expression of BBC3 and axonal degeneration in human iPSC-MN (A) Paradigm illustrating experimental approach. (B) Representative pictures of betaIII-Tubulin-positive neurons (magenta) with NPM1 signal (LUT, blue = low fluorescence intensity, white = high fluorescence intensity) in the nucleus (white) of UKERiff2-X-18. Scale bar: 10 μm. (C) Bar plot quantifying fluorescence intensity ratio of NPM1 in non-nucleolar nucleoplasm/nucleolar area (*n* = 4 cell lines). Dots depict individual values per cell line, graph as median with interquartile range. Statistics: Friedman test: *p* value: 0.0062. Dunn’s multiple comparison test: ∗ < 0.05; ∗∗ < 0.01; ∗∗∗ < 0.001. Significance in post-hoc analysis only found between DMSO and 1,000 nM. (D) Western blot of BBC3 (also known as PUMA) in iPSC-MN of 5 cell lines treated for 5 days with DMSO, 100 nM, and 1,000 nM branaplam. GAPDH serves as loading control. (E) Bar plot showing densitometric quantification of BBC3 signal normalized to GAPDH. Dots depict individual values per cell line, graph as median with interquartile range. Statistics: Friedman test: *p* value: 0.0008. Dunn’s multiple comparison test: ∗ < 0.05; ∗∗ < 0.01; ∗∗∗ <0.001. Significance in post-hoc analysis only found between DMSO and 1,000 nM.

Multiple cellular dysregulations result in p53 activation. Since branaplam is an RNA-targeting molecule, we reasoned that an RNA-associated mechanism is likely. Here, nucleolar stress is a prominent example: Dysregulation of rRNA transcription or metabolism in conjunction with other mechanisms disrupts the nucleolar structure, resulting in p53 activation. First, we investigated whether branaplam alters rRNA biogenesis, a potential inducer of nucleolar stress and p53 signaling. Branaplam-treated fibroblasts exhibit higher levels of unprocessed, high molecular rRNA precursors, indicating an impact on rRNA processing (Figures S3A and S3B). Nucleolar stress can be measured via the translocation of NPM1 from the nucleolus to the nucleoplasm.^22^ We explored whether this is present in human neurons upon branaplam exposure (Figure 3A). iPSC-MN treated with 1,000 nM of branaplam for 5 days exhibited increased ratios of NPM1 signal in the nucleoplasm vs. the nucleolus (Figures 3B and 3C). This indicates the induction of nucleolar stress in iPSC-MN. P53-mediated BBC3 expression can induce neurite degeneration.^12^^,^^13^^,^^14^^,^^23^ We observed an increased BBC3 expression in the iPSC-MN treated with branaplam, indicative of activated p53 signaling (Figures 3D, 3E, and S3C).

## Discussion

Our results indicate that this novel type of small molecule may have unknown off-targets that lead to clinically relevant side effects in adult patients, which was previously not noted. Branaplam has been first under investigation in infants with SMA without severe adverse effects. However, the development for SMA patients was discontinued due to three alternative options for causal SMA treatment.^24^ A low dose, short treatment paradigm did not alter the proliferation of human cortical progenitor cells or induce apoptosis in human cortical neurons,^3^ and orally administered branaplam was also reported to have no impact on neurogenesis in the CNS in juvenile mice, rats, and dogs.^25^ Our straightforward *in vitro* framework suggests clear neuropathogenic effects, including detecting elevated levels of NfL, a clinically accepted biomarker for neurite degeneration independent of HD genotype. This effect was observed in a more elaborate dog model upon branaplam administration over several weeks.^26^ This reflects the severe affection of the peripheral nervous system and specifically axons as seen in the VIBRANT-HD trial leading to its termination. Multiple compound-related effects may act synergistically and explain the discrepancy between the infancy SMA and the adult HD trials. The pharmacokinetic profile of infants vs. adults may be different. Also, more subtle peripheral neuropathic effects, and especially motor neuropathy, may have been missed in infants with SMA due to the disease phenotype of motor neuron degeneration versus potential degeneration of the motor axon due to a drug’s side effect. Potential beneficial effects on motor neurons by branaplam due to elevating SMN2 levels may exceed the toxic changes induced by the drug as a side effect on the same neurons. To finally resolve this issue, more detailed investigations will be necessary. They will require in-depth analyses and comparison of the clinical parameters of the branaplam trials in SMA infants (NCT02268552) and HD patients (NCT05111249).

On a molecular level, these side effects may arise due to mRNA-independent mechanisms of branaplam. Multiple drugs lead to peripheral neuropathy as a side effect, most prominently chemotherapeutics such as taxans and platin-containing substances. While taxans are thought to destabilize microtubules directly, platin-containing chemotherapeutics exhibit manifold cytotoxic effects.^27^ Oxaliplatin has been shown to induce nucleolar stress, as measured via translocation of NPM1 from the nucleolus to the nucleoplasm^23^^,^^24^^,^^25^ and p53 stabilization^5^ by disrupting rRNA biogenesis,^28^^,^^29^ as observed in the present study. Therefore, off-target effect mechanisms on non-coding RNAs may be involved and have to be considered, especially for drugs known to bind and interfere with RNA. However, it is important to note that multiple molecular mechanisms besides p53-signaling may be involved in the pathogenesis of branaplam-induced neuropathy.

Data from the VIBRANT-HD trial published on clinicaltrials.gov (NCT05111249) suggest increased serum NfL in 76.2% of individuals dosed orally for 17 weeks with once-weekly 56 mg branaplam. Pharmacokinetic data indicate a maximal exposure of patients in plasma (C_max_) of 45.3 ng/mL and a weekly plasma exposure of 3190 h∗ng/mL (AUC_168h_). In our *in vitro* system, we observe clear effects after 5 days regarding NfL increase at a concentration of 1,000 nM and partial trends regarding neurite disintegrity already at 100 nM. Assuming the compound is stable *in vitro*, 1,000 nM corresponds to a C_max_ of 393.5 ng/mL and an AUC_120h_ 47220 h∗ng/mL. Our calculations suggest a ∼8.7x higher C_max_ and ∼14.8x higher AUC in our *in vitro* system compared to a 1-week *in vivo* concentration. While our concentrations *in vitro* are higher, it is essential to note that patients were exposed to the drug ∼23 times longer than in our *in vitro* model. While the cumulative dose used in this study that elicited a robust NfL increase is in line with clinical pharmacokinetic data, we cannot rule out the possibility of a confounding contribution of suprapharmacological peak doses to the observed effects *in vitro*. Further investigations are warranted to delineate the contribution of dose-dependent effects. Modeling pharmacokinetic dynamics *in vitro* is challenging, and the dynamics of concentration changes due to uptake and elimination are not reflected, so further investigations are needed. However, our data indicate that clinically described axonal degeneration corresponds to the *in vitro* observations.

Due to the nucleic acid sequence and structure-dependent mechanisms, the present study illustrates the power of human stem cell-derived *in vitro* models for testing on- and off-target effects of nucleic acid-targeting drugs as a complementary system to existing preclinical models.

### Limitations of this study

This study employed an *in vitro* based approach to explore pharmacological adverse effects. It is important to acknowledge that it may not completely recapitulate the complexities of *in vivo* situation, particularly regarding pharmacokinetics, and here especially the dynamics related to absorption, metabolization, and elimination. Additionally, the drug exposure duration in our *in vitro* experiment is shorter (23 times) compared to the in the clinical trial setting.

## Resource availability

### Lead contact

Requests for further information and resources should be directed to and will be fulfilled by the lead contact, Prof. Dr. med. Beate Winner (beate.winner@fau.de).

### Materials availability

Materials that support the findings of this study are available from the corresponding author upon reasonable request.

### Data and code availability

• Count tables of fibroblasts and iPSC-neurons treated without and with branaplam and branaplam’s targets from our previous study were used, and the respective counts are publicly available in the source data of that paper (https://doi.org/10.1038/s41467-022-34419-x).^3^
• Gene lists associated with hereditary neuropathy were acquired from gene panel diagnostics from the Mayo Clinic Laboratories (“Peripheral Neuropathy Expanded Panel”; list from 2019; https://www.mayocliniclabs.com/) and the MGZ Munich (“Neuropathy/motor neuropathy comprehensive panel”, list available in November 2022, https://www.mgz-muenchen.com/).
• The p53 ChIP-seq dataset (ENCFF488WQN) was downloaded from the ENCODE project.^19^^,^^20^
• No original code was generated in this study.
• Any additional information required to reanalyze the data reported in this paper is available from the lead contact upon request.

## Acknowledgments

We thank Naime Zagha, M.Sc., Sonja Ploetz, and Michaela Farrell for excellent technical support. Access to the MESO QuickPlex system was kindly provided by the Translational Radiobiology (Department of Radiation Oncology, University Hospital Erlangen). Data analysis was performed at the servers hosted by the “Data Integration Center” (DIC) and accommodated by Dr. Wolfgang Krebs and Dr. Pooja Gupta within the “Core Unit for Bioinformatics, Data Integration and Analysis” (CUBiDA), Universitätsklinikum Erlangen, Erlangen, Germany.

Funding came from the 10.13039/501100001659German Research Foundation, DFG (WI 3567/2-1 (B.W.); GRK2162/270949263 (B.W. and J.W.), CRU5024/505539112 WI 3567/4-1 (B.W.) and WI 1620/4-1 (J.W.), the TreatHSP consortium (BMBF 01GM1905B, 01GM2209B to B.W., M.R., and J.W.) and ACS iIMMUNE (BMBF 01EO2105 to B.W. and M.R.), the Bavarian Ministry of Science and the Arts in the framework of the ForInter network (B.W. and J.W.), the Interdisciplinary Center for Clinical Research (IZKF) 10.13039/501100018935Universitätsklinikum Erlangen (ELAN-Fond P153 (F.K.), Advanced project [E30] (B.W. and J.W.) and Universitaetsstiftung Medizin (J.W.) and the 10.13039/501100003042Else Kröner-Fresenius-Stiftung (2024_EKEA.50) (F.K.).

## Author contributions

Conceptualization, F.K., T.B., J.W., and B.W.; formal analysis, F.K.; investigation, F.K., T.B., S.N., and L.K.; resources, M.R., J.W., and B.W.; funding acquisition, F.K., J.W., B.W., and M.R.; supervision J.W. and B.W.; writing – original draft, F.K. and T.B. made figures and wrote the original draft of the manuscript; writing – review and editing, F.K., T.B., M.R., J.W., and B.W.

## Declaration of interests

Full financial disclosures of all authors for the previous 12 months.

F.K.: Employment (University Hospital Erlangen); T.B.: Employment (University Hospital Erlangen); S.N.: Employment (University Hospital Erlangen); L.K.: Employment (University Hospital Erlangen); M.R.: Employment (University Hospital Erlangen, Klinikum Forchheim), grants (BMBF, Förderverein für HSP-Forschung), honoraria (Orphalan, Desitin, Ever Pharma, Zambon, Bial, DGN), prize (Euro-HSP).; J.W.: Employment (University Hospital Erlangen), honoraria: Zambon, Bial, UCB.; B.W.: Employment (University Hospital Erlangen).

## Declaration of generative AI and AI-assisted technologies in the writing process

During the preparation of this work the authors used Grammarly in order to improve for correct spelling and grammar and plagiarism. After using this tool, the authors reviewed and edited the content as needed and take full responsibility for the content of the publication.

## STAR★Methods

### Key resources table

**Table:** 

REAGENT or RESOURCE	SOURCE	IDENTIFIER
**Antibodies**		

Nucleophosmin	abcam	Cat# ab10530; RRID: AB_297271
beta-III-Tubulin	abcam	Cat# ab18207; RRID: AB_444319
TAU	Santa Cruz Biotechnology	Cat# sc-1995; RRID: AB_632467
ISL1	DSHB	Cat# 39.4D5-s; RRID: AB_2314683
BBC3 (PUMA)	abcam	Cat# ab9643; RRID: AB_296537
GAPDH	Millipore	Cat# CB1001; RRID: AB_2107426
donkey anti-mouse IgG (H + L) secondary antibody, Alexa Fluor 488 conjugate	Thermo Fisher Scientific	Cat# A-21202; RRID: AB_141607
donkey anti-goat IgG (H + L) secondary antibody, Alexa Fluor 546 conjugate	Thermo Fisher Scientific	Cat# A-11056; RRID: AB_142628
donkey anti-Rabbit IgG (H + L) Secondary Antibody, Alexa Fluor 647 conjugate	Thermo Fisher Scientific	Cat# A-31573; RRID: AB_2536183
donkey anti-mouse IgG (H + L) secondary antibody, HRP	Thermo Fisher Scientific	Cat# SA1-100; RRID: AB_325993
Donkey anti-rabbit IgG (H + L) secondary antibody, HRP	Thermo Fisher Scientific	Cat# SA1-200; RRID:AB_325994

**Chemicals, peptides, and recombinant proteins**		

TriZOL	Invitrogen	21267501
DNase/RNase-free H2O	Invitrogen	10977015
RNA loading buffer	NEB	B0363S
20% PFA	Electron Microscopy Sciences	15713-1L
GelRed	Carl Roth	0984.1
positively-charged nylon membrane	Roche	11209299001
ULTRAhyb-Oligo buffer	Invitrogen	AM8663
SDS	Carl Roth	8029.3
ECL solution	Amersham	GERPN2106
BD Perm/Wash Buffer	BD Bioscience	554723
BD Fixation/Permeabilization	BD Bioscience	554714
Triton X-100	Invitrogen	X100-100ML
DMSO	Merck	D8418
DMEM/F12 + Glutamax	Thermo Fisher Scientific	31331028
Neurobasal	Thermo Fisher Scientific	21103049
B27	Thermo Fisher Scientific	17504044
N2	Thermo Fisher Scientific	17502048
Ascorbic acid	Sgima	A4544-25G
Retinoic acid	Sigma-Aldrich	R2625
Smoothed agonist	Tocris	4366
Y-27632	Tocris	1254
brain-derived neurotrophic factor	Peprotech	450–02
glial-derived neurotrophic factor	Peprotech	450–10
ciliary neurotrophic factor	Peprotech	450–13
Geltrex	Thermo Fisher Scientific	A1413302
DAPT	TOCRIS	2634/10
PBS (w/o CaCl2, w/o MgCl2)	Thermo Fisher Scientific	14190144

**Critical commercial assays**		

EdU Click FC ROTIkit for Flow Cytometry Alex Fluor 594	Carl Roth	7781.1
R-PLEX Human Neurofilament L Antibody Set	Meso Scale Discovery system	F217X
U-Plex® Development Pack, 96-well 2-Assay SECTOR Plate kit	Meso Scale Discovery system	K1522N

**Deposited data**		

RNAseq dataset	An alternative splicing modulator decreases mutant HTT and improves the molecular fingerprint in Huntington’s disease patient neurons	https://doi.org/10.1038/s41467-022-34419-x
p53 ChIP-seq dataset	ENCODE project	ENCFF488WQN

**Experimental models: Cell lines**		

UKERf33Q	University Hospital Erlangen	Ctrl
UKERfB26	University Hospital Erlangen	Ctrl
UKERf4CC	University Hospital Erlangen	Ctrl
UKERf4L6	University Hospital Erlangen	Ctrl
UKERf4Q4	University Hospital Erlangen	HD
UKERfOP5	University Hospital Erlangen	HD
UKERf59H	University Hospital Erlangen	HD
UKERf919	University Hospital Erlangen	HD
UKERiff2-X-18	University Hospital Erlangen	Ctrl
UKERiff2-SC-11	University Hospital Erlangen	Ctrl
UKERi33Q-E−2	University Hospital Erlangen	Ctrl
UKERi33q-E−6	University Hospital Erlangen	Ctrl
CV-B	Coriell	Ctrl

**Software and algorithms**		

CytExpert Software 2.4	Beckman Coulter	N/A
Prism 9	GraphPad	N/A
DESeq2	Love et al.^30^	N/A
Python	Python Software Foundation	N/A
Fiji	Schindelin et al.^31^	N/A

### Experimental model and study participant details

The generation and use of these human cell lines were approved by the Institutional Review Board (Nr. 4120 and 259_17B: Generierung von humanen neuronalen Modellen bei neurodegenerativen Erkrankungen). We used 8 previously published fibroblast and iPSC lines from 4 Ctrl (UKERf33Q (sex: f; age at biopsy: 45; ethnicity: caucasian), UKERfB26 (sex: m; age at biopsy: 43; ethnicity: caucasian), UKERf4CC (sex: m; age at biopsy: 52; ethnicity: caucasian), UKERf4L6 (sex: m; age at biopsy: 32; ethnicity: caucasian)) donors and 4 individuals with HD (UKERf4Q4 (sex: f; age at biopsy: 44; ethnicity: caucasian), UKERfOP5 (sex: m; age at biopsy: 54; ethnicity: caucasian), UKERf59H (sex: m; age at biopsy: 26; ethnicity: caucasian), UKERf919 (sex: f; age at biopsy: 23; ethnicity: caucasian)) 3. We also used 5 previously published iPSC-derived motor neuron progenitor lines originating from 3 Ctrl individuals (UKERiff2-X-18 (sex: m; age at biopsy: 26; ethnicity: caucasian), UKERiff2-SC-11 (sex: m; age at biopsy: 26; ethnicity: caucasian), UKERi33Q-E-2 (sex: f; age at biopsy: 45; ethnicity: caucasian), UKERi33q-E-6 (sex: f; age at biopsy: 45; ethnicity: caucasian), CV-B (sex: m; age at biopsy: N/A; ethnicity: caucasian)).^5^ All cell lines were tested regullary for mcyoplasm contamination.

### Method details

#### Differential gene expression analysis, overlaps and transcription factor binding site prediction

Differential gene expression was performed using DESeq2 (1.34.0)^30^ for fibroblasts (n = 8 DMSO (n = 4 Ctrl, n = 4 HD); n = 8 branaplam (n = 4 Ctrl, n = 4 HD)) and iPSC-neurons (n = 6 DMSO (n = 3 Ctrl, n = 3 HD); n = 6 branaplam (n = 3 Ctrl, n = 3 HD). A significant threshold was set to an adjusted P value of below 0.05. The overlaps of gene lists (branaplam targets, two neuropathy gene panel lists, differentially expressed genes in fibroblasts and neurons) was performed in Python and visualized as Venn diagrams using the matplotlib plugin. Counts were transformed into RPKM (Reads Per Kilobase of transcript per Million reads mapped) values and the fold change of expression upon was calculated first for each individual and then averaged across the group (Ctrl or HD sample). Fold changes were plotted as cumulative distribution plots in Python using the ecdfplot function of the seaborn plugin. Significant differences were determined using 2 sample KS-test using scipy.stats. As a control fold change values of a shuffled list of genes with equal size as the tested condition was used. To predict enrichment of transcription factor binding sites (TFBS) in differentially expressed genes, up- and downregulated genes were plugged into the DAVID gene ontology server^32^ and investigated for enrichment of UCSC TFBS. As a background, we uploaded all genes expressed in our fibroblast dataset, defined as a mean RPKM >0.5 of a gene across all samples.

#### P53 ChIP-seq validation

For validation of p53 association to gene expression changes, a p53 ChIP-seq peak dataset (conservative IDR thresholded peaks) was downloaded from ENCODE (ENCFF488WQN). We considered a region of 5000 bp upstream of the gene start (determined via gencode annotation version 26) as a region of interest for putative promoter regions using pybedtools intersect (u = True). Fold changes of the determined putative p53 targets were plotted as cumulative distribution plots in Python using the ecdfplot function of the seaborn plugin. Significant differences were determined using 2 sample KS-test using scipy.stats. As a control, values of a shuffled list of genes with equal size as the tested condition was used.

#### Fibroblast culture

Fibroblasts were resuspended in fibroblast growth medium (FGM; 75% DMEM, 15% FCS, 2 mM l-glutamine, 100 μg/mL penicillin/streptomycin, 2 ng/mL fibroblast growth factor 2) and plated on polystyrene cell culture flasks. Medium was changed twice a week. To expand the fibroblasts, they were split by washing the cells once with PBS (w/o CaCl2, w/o MgCl2), followed by incubation with Trypsin at 37°C for 20 min. The detached cells were collected in DMEM, transferred to a centrifugation tube and centrifuged for 5 min at 300 ×g at room temperature. Afterwards, the supernatant was removed, cells were resuspended in FGM and plated on a new polystyrene cell culture flask. For imaging-based experiments, fibroblasts were seeded on a 96-well glass bottom imaging plate (1000 cells per well). The cells were treated with three different concentrations of branaplam (treatment concentrations: 10 nM, 100 nM and 1000 nM) as well as the vehicle (DMSO). For flow cytometry, cells were seeded on a 10 cm dish (200,000 cells per dish) and treated with branaplam (10 nM, 100 nM and 1000 nM), the vehicle (DMSO) and paclitaxel for 72 hours. All treatments were performed for 72 h, with complete media changes every 24 h.

#### Motor neuron differentiation

A 2D dual smad based differentiation protocol was used to generate motor neurons (MN) from hiPSC as published before.^5^ Briefly, iPSC were dissociated into single cells using Accutase. After cell counting, 2 × 10^5^ cells per cm^2^ were plated on a Matrigel-coated well (17.5 μg/cm^2^ of Matrigel in DMEM/F12) with mTESR PLUS supplemented with 5 μM Y-27632 (RI). Around 24 h later, the cells reached 95% confluency. For the differentiation into MN, a basal-media, further referred as N2B27 (DMEM/F12, 1 × 2, 1xB27, 100 μM ascorbic acid, 1xPen/Strep), was used for maintenance and dilution of compounds. From day one to day six, the cells were incubated with N2B27 supplemented with 1 μM Dorsomorphin (Dorso), 10μM SB431542 (SB), 3μM CHIR99021 (CHIR). From day 3 on, 5 μM RI were supplemented and the media volume was gradually increased (0.2 mL/cm^2^ on days one and two, 0.3 mL/cm^2^ on days 3 and 4, and 0.4 mL/cm^2^ on the following days). The media was exchanged on daily basis. From day six to day 15 the cells were incubated with 0.5xN2B27 with Dorso, SB and, in addition, 1.5 μM retinoic acid (RA) and 200 nM of Smoothed agonist (SAG) and 5 μM RI with 0.416 mL/cm^2^. When the cells reached the stage of MN progenitors (MNPs, day 15 of differentiation), the MNPs were dissociated into single cells using Accutase. The single cells were resuspended in 1 mL of a N2B27 supplemented with 1.5 μM retinoic acid and 200 nM of Smoothed agonist (SAG), 10 μM Rock inhibitor and 2 ng/mL of brain-derived neurotrophic factor (BDNF), glial-derived neurotrophic factor (GDNF), and ciliary neurotrophic factor (CNTF), respectively. This combination of growth factors will be referred to as neurotrophic factors (NFs) further on. 35,715 cells/cm^2^ were seeded on Geltrex-coated 12-well plates and cultured with 1 mL media per well. Two and 4 days after seeding, media was exchanged completely and the cells were fed with N2B27 and 1.5μM RA, 200 nM SAG, 10 μM RI and NFs. From day 6 on, cells were cultured in N2B27 and 2 μM DAPT, 10 μM RI and NFs. On day 7 after seeding, an additional feeding step was performed with cold N2B27 and 2 μM DAPT, 10 μM RI and NFs plus Geltrex and 0.5 μg/mL laminin to enhance cell attachment. On day 9, the cells were washed 1x with DMEM/F12 + Glutmax and then fed further on with N2B27 and 10 μM RI, NFs and 0.5 μg/mL laminin till the end of differentiation (day 14 after seeding). For imaging-based experiments, 6 days after seeding cells were dissociated with Accutase, and plated on Geltrex coated cover slips in 24-well plates (50,000 cells per well) or 96-well imaging plates (25,000 cells per well). Branaplam treatment (10 nM, 100 nM, 1000 nM) or vehicle (DMSO) was administered to the cells on day 9, 10 and 12 after seeding by exchanging the media completely.

#### MSD® multi-spot assay system

Multiplex ELISA was performed using the Meso Scale Discovery® system (MSD®; Rockville, MD, USA) according to the manufacturer’s recommendation. For this experiment the U-Plex® Development Pack, 96-well 2-Assay SECTOR Plate kit (Cat.: K1522N) was used together with the R-PLEX Human Neurofilament L Antibody Set (Cat.: F217X) and recommended buffers, Diluent 11 (Cat.: R55BA) and Diluent 12 (Cat.: R50JA). For the analysis, cell culture supernatants were diluted 1:2 in Diluent 12. All steps were performed according to the manufacturer’s instructions. Provided plates were coated with Linker-coupled antibodies one day before the assay. The biotinylated antibody was combined with the assigned Linker (Linker 1 for NF-L) and adjusted to 6 mL with the provided Stop Solution. 50 μL of the coating solution was added to each well and the plate was subsequently incubated for 1 h at RT and afterwards washed three times with PBS 0.05 % Tween-20. The plate was stored at 4°C over night. For the assay, provided calibrators were diluted in a 4-fold serial dilution using Diluent 12 to generate eight standards. Next, 25 μL of Diluent 12 was added to each well, followed by 25 μL of prepared calibrator standards or sample dilutions. All standards and samples were added in duplicates. The plate was incubated at RT with shaking for one hour. After the plate was washed three times as described above, 50 μL of detection Antibody Solution was added to each well. The 100X stock solution of the detection antibody was diluted in Diluent 11 shortly before. After an incubation for 1 h, the plate was again washed three times and 150 μL of provided MSD GOLD Read Buffer B was added to each well. The measurement was performed by MESO® QuickPlex® SQ 120MM (Cat.: Al1AA) and subsequent analysis was carried out using MSD® Discovery Workbench® Version 4.0. All measured concentrations used for further analysis were within the manufacturer’s recommended detection range.

#### Immune fluorescence imaging

Cells were fixed in 4 % paraformaldehyde (PFA) for seven minutes at room temperature and subsequently washed 3x with PBS for 3 mins. A total of five different Ctrl lines were processed in parallel. The cells were permeabilized and blocked in 0.3 % Triton X-100 and 5 % donkey serum in PBS for 30 mins at room temperature. Afterwards, the cells were incubated with primary antibodies (Nucleophosmin: ab10530, Abcam, 1:250; beta-III-Tubulin: ab18207, Abcam, 1:200; TAU: sc-1995, Santa Cruz Biotechnology, 1:500; ISL1: 39.4D5-s, DSHB, 1:250) in 5% donkey serum in PBS at 4°C overnight. After washing twice with PBS for 3 mins, incubation with secondary antibodies (donkey anti-mouse IgG (H+L) secondary antibody, Alexa Fluor 488 conjugate: Thermo Fisher Scientific, A-21202, 1:500; donkey anti-goat IgG (H+L) secondary antibody, Alexa Fluor 546 conjugate: Thermo Fisher Scientific, A-11056, 1:500; donkey anti-Rabbit IgG (H+L) Secondary Antibody, Alexa Fluor 647 conjugate: Thermo Fisher Scientific, A-31573, 1:500) for 1 h at room temperature was performed. Then cells were washed once with 0.1 % Triton X-100 and 5 % donkey serum in PBS, followed by the nuclei staining using 0.2 μg/mL DAPI in PBS. Cells were washed twice with PBS, followed by mounting the cover split on the object slide using Mowiol solution. Imaging was performed with a Zeiss Observer.Z1, including Apotome technology.

#### NPM1 staining analysis

Images were taken using a Zeiss Observer.Z1 using Apotome with a 63x objective. For each condition and line, 20 fields of view were imaged using 29 slices (14 μm) Z-stacks. After Apotome correction, a maximum intensity projection was applied, the images were exported as tiff files and analyzed using CellProfiler. Nuclei and neurons were identified using IdentifyPrimaryObjects with the DAPI and beta-III-Tubulin images (DAPI: global Otsu thresholding with 2 classes, smoothing scale: 0.5, correction factor: 2; beta-III-Tubulin: adaptive Otsu thresholding with 2 classes, smoothing scale: 1.3488, correction factor: 1.3) and nuclei that belong to neurons were determined (RelateObjects). The NPM1 image was masked for areas only belonging to neuronal nuclei (MaskImage), enhanced (EnhanceEdges, Sobel method) and a gaussian filter was applied (Sigma: 2.5). Next, primary objects (nucleoli) were determined using IdentifyPrimaryObjects (adaptive Otsu thresholding with 2 classes, smoothing scale: 1, correction factor: 1, do not allow filling holes in objects), merged into a single object (SplitOrMergeObjects) and holes within Objects are filled (FillObjects, maximum hole size: 50). The nucleoplasm area (non-nucleolar nucleus area) was identified using IdentifyTertiaryObjects. The MeasureObjectSizeShape and MeasureObjectIntensity were subsequently applied. A ratio of NPM1 fluorescent intensity in the nucleoplasm that is not the nucleolus (non-nucleolar) vs. the nucleolar area was calculated for each cell. The median for this ratio was calculated for each line and condition and used for statistical analysis.

#### Neurite disintegration analysis

Images were taken using a Zeiss Observer.Z1 using Apotome with a 20x objective. For each condition and line, three to four fields of view were imaged using 50 slices (36,75 μm) Z-stacks. After Apotome correction, a maximum intensity projection was applied and the images were exported as a tiff file.

Quantification of neurite disintegration was performed on basis of the quantification of the microtubule depolymerization index.^12^ The analysis was performed in CellProfiler (4.1.3). The analysis is based on that destabilized microtubules such as in damaged neurites and neurite swellings occupy higher intensity values. In a first step, the intensity and standard deviation of intensity in control conditions (DMSO) within neurites is determined to set an intensity threshold (3 standard deviations above the mean intensity) to determine occupancy of pixels to the highest intensities in neurite occupying areas. This threshold, determined for each cell line separately, is then applied to all images and the area occupied by disintegrated neurite areas is divided by the total neurite area in an image and defined as the neurite disintegration index.

Specifically, the original input image was subject to the EnhanceOrSupressFeatures function to enhance neurites (Feature type: Neurites; Enhancement method: Line structures; Feature size: 10). This was used to determine a binarized object of interest (neurite area) using the IdentifyPrimaryObjects function (Threshold strategy: Global; Threshold method: Otsu with two classes; Smoothing scale: 0; Threshold correction factor: 1.3; no filling of holes in objects) and merged into a single object (SplitOrMergeObjects). The fluorescent intensity and standard deviation thereof in this area was quantified (MeasureObjectIntensity). For each cell line, the results from the DMSO images were averaged separately to determine a cell line-specific intensity threshold (3 standard deviations above the mean intensity). In a second step, all images were processed from the start again in the same fashion as described before. A mask of the binary object of interest (neurite area) was applied to the original beta-III-Tubulin image. The cell line specific intensity threshold was applied to the masked image to obtain a binary image of disintegrated neurite areas (IdentifyPrimaryObject; Threshold strategy: Global; Threshold smoothing scale: 0) and merged into a single object per image (SplitOrMergeObjects). The area occupied by the total neurite area and the disintegrated area was determined (MeasureObjectSizeShape) and exported. A disintegrated neurite to total neurite area ratio was applied for each image (AreaShape_Area columns used) and averaged for each cell line.

#### Western blot

For whole cell lysates, cells were lysed in Radio-immunoprecipitation buffer (RIPA: buffer ingredients: 50mM Tris-HCl (pH7.4), 1% NP-40, 0.5% Deoxycholic acid sodium salt, 0.1% Sodium dodecyl Sulfate (SDS), 150mM Sodium chloride, 2mM Ethylenediaminetetraacetic acid (EDTA), 50mM Sodium fluoride) followed by sonication with a Diagenode Bioruptor Pico (setting: 30s ON, 30s OFF, 5 cycles, high frequency). The protein concentration was estimated by bicinchoninic acid (BCA) assay and equal concentrations were applied. All immunoblots were run on 4–12% Bis-Tris gels with NuPAGE MOPS running buffer for 90 min at 150 V. Proteins were transferred to a PVDF membrane with 10% methanol in NuPAGE transfer buffer at 10 V for 14 h at 4°C. The membrane was then blocked for 1 h in 5% dry milk in TBS-T. Afterwards, primary antibodies (BBC3 (PUMA): ab9643, Abcam, 1:500; GAPDH: CB1001, Millipore, 1:5000) in 5% dry milk in TBS-T were incubated for 1 h at room temperature. Subsequently, the membrane was washed twice for 3 mins in TBS-T and then incubated with the secondary HRP-conjugated antibody (donkey anti-mouse IgG (H+L) secondary antibody, HRP, Thermo Fisher Scientific, SA1-100, 1:5000; Donkey anti-rabbit IgG (H+L) secondary antibody, HRP, Thermo Fisher Scientific, SA1-200, 1:5000) in 5% dry milk in TBS-T for 1 h at room temperature. The membrane was washed three times for 3 mins with TBS-T and incubated in the dark with ECL solution. The chemiluminescent signal was detected using a film and developed in the dark with various exposure times. Western blot signals were quantified densitometrically using Fiji. The signal was normalized to the corresponding signal of GAPDH.

#### Northern blot

TriZOL was used for RNA extraction according to the manufacturer’s instructions. All steps were performed at 4°C. The RNA was resuspended in DNase/RNase-free H2O. 1.5 μg of RNA was diluted in 1x RNA loading buffer (NEB) with 0.4 M PFA (20% PFA, Electron Microscopy Sciences) to a total volume of 20 μL and incubated at 70°C for 5 min for denaturation. Samples and 5 μL RNA ladder (Thermo Scientific) were loaded in casted RNAse-free 0.8% agarose gel (1x TT buffer, 1:10000 GelRed, 0.4 M PFA). The gel was run in 1x TT buffer (1.5 M Tricine, 1.5 M triethanolamine) at 100 V for 60 min. RNA separation was verified by detecting GelRed signal using ChemiDoc (BioRad). Next, RNA was transferred from the gel into a positively-charged nylon membrane (Roche) in 0.5x TT buffer at 0.2 A for 1 h 10 min. After washing the membrane once with MilliQ water, RNA was crosslinked to the membrane with UV with ChemiDoc (BioRad) for 3 min. To control if the transfer was successful, the RNA on the membrane was visualized with ChemiDoc (BioRad). The blot was prehybridized in ULTRAhyb-Oligo buffer (Invitrogen) for 15 min at 60°C and 40 rpm. It was then hybridized overnight at 60°C and 40 rpm with 5 nM of the end-labeled oligonucleotide (ITS1, ITS2, and 5′ETS) in ULTRAhyb-Oligo buffer. The blot was washed in stringency wash buffer (2X SSC 300 mM NaCl, 30 mM sodium citrate, pH 7.0, Life Technologies; 0.5% SDS) three times at 60°C. Before detection, the membrane was washed twice for 5 min in 1x washing buffer (1x PBS, 2.5% SDS buffer), then washed twice in 1x blocking solution (1x PBS, 0.5% SDS, and 3% BSA). The membrane was blocked for 45 min in 1x blocking solution. The incubation of the membrane was performed with conjugate solution (10 mL blocking buffer plus 1 μl of streptavidin-HRP conjugate) for 30-45 min at RT and followed by washing in 1x blocking buffer and three times in 1x washing buffer for 15 min. Last, the membrane was washed twice in 1x PBS for 2 min each. The membrane was covered with ECL solution (Amersham) for one minute for chemiluminescence detection. The signal was detected with MP ChemiDoc (BioRad).

#### Cell cycle assay

For cell cycle analysis, human fibroblasts were cultured and treated as described before. For EdU incorporation, culture media was supplemented with 10 μM 5-Ethyl-2′-deoxyuridine (EdU) for one hour at 37°C. Cells were immediately dissociated after incubation as described before and resuspended in FC buffer (2 % FCS, 0.01 % sodium azide, 3 mM EDTA). The suspension was dispensed into 5 mL tubes (Sarstedt) at 5x10E5 cells per tube. Cells were fixed and permeabilized in 100 μL BD Fixation/Permeabilization Solution (BD Bioscience) for 10 min. Then 1 mL of BD Perm/Wash Buffer (BD Bioscience) supplemented with 0.1% Triton X-100 was added to and cells were centrifuged at 1,700 rpm for 3 min. EdU labeling was performed with EdU Click FC ROTI®kit for Flow Cytometry (Carl Roth) according to the manufacturer protocol. EdU-labeled cells were stained with the click assay cocktail for 30 min at room temperature, protected from light. Subsequently, 1 mL of BD Perm/Wash Buffer (BD Bioscience) supplemented with 0.1 % Triton X-100 was added and cells were centrifuged at 1,700 rpm for 3 min. For DNA staining, cells were resuspended in 1 mL FC buffer supplemented with 2 μg/mL DAPI. After 30 min incubation at RT, protected from light Flow cytometry was performed with Cytoflex S. Sample flow rate was adjusted to a maximum count of 400 events per second for singlet counting. Analysis was performed with CytExpert Software. Cell cycle phases were gaited from gaited single nuclei events, according to their DNA content (2n = G_0_/G_1_, 4n = G_2_/M) or EdU positive events (S-Phase) (Figure S2).

### Quantification and statistical analysis

GraphPad Prism 9 was used to visualize data and calculate statistics for pairwise and grouped analyses (RPKM analysis of specific genes, FACS quantification, densitometric quantification of western blot, image data analyses). DMSO samples and their respective treated samples were considered as paired. When comparing two conditions, Mann–Whitney–U test was used. When comparing multiple groups (e.g., different branaplam concentrations), Friedman test was used for non-normally distributed data with Dunn’s post hoc test, respectively, to identify differences between individual groups. For grouped analyses (e.g. DMSO vs. branaplam in Ctrl vs. HD), paired two-way ANOVA was used. The statistical test and exact *p* value used for calculating the significance of each graph is indicated in the figure legend. A *p* value ≤ 0.05 was considered as significant. When *p* values were depicted as asterisks the following applies: ∗ <0.05; ∗∗ <0.01; ∗∗∗ <0.001.
