# Supplementary material for: RNA splicing modulator for Huntington’s disease treatment induces peripheral neuropathy
Source: iScience. 2025 Apr 8;28(5):112380. doi: 10.1016/j.isci.2025.112380 (PMC12059699; doi:10.1016/j.isci.2025.112380)
Supplement: Document S1. Figures S1–S3 [file mmc1.pdf]

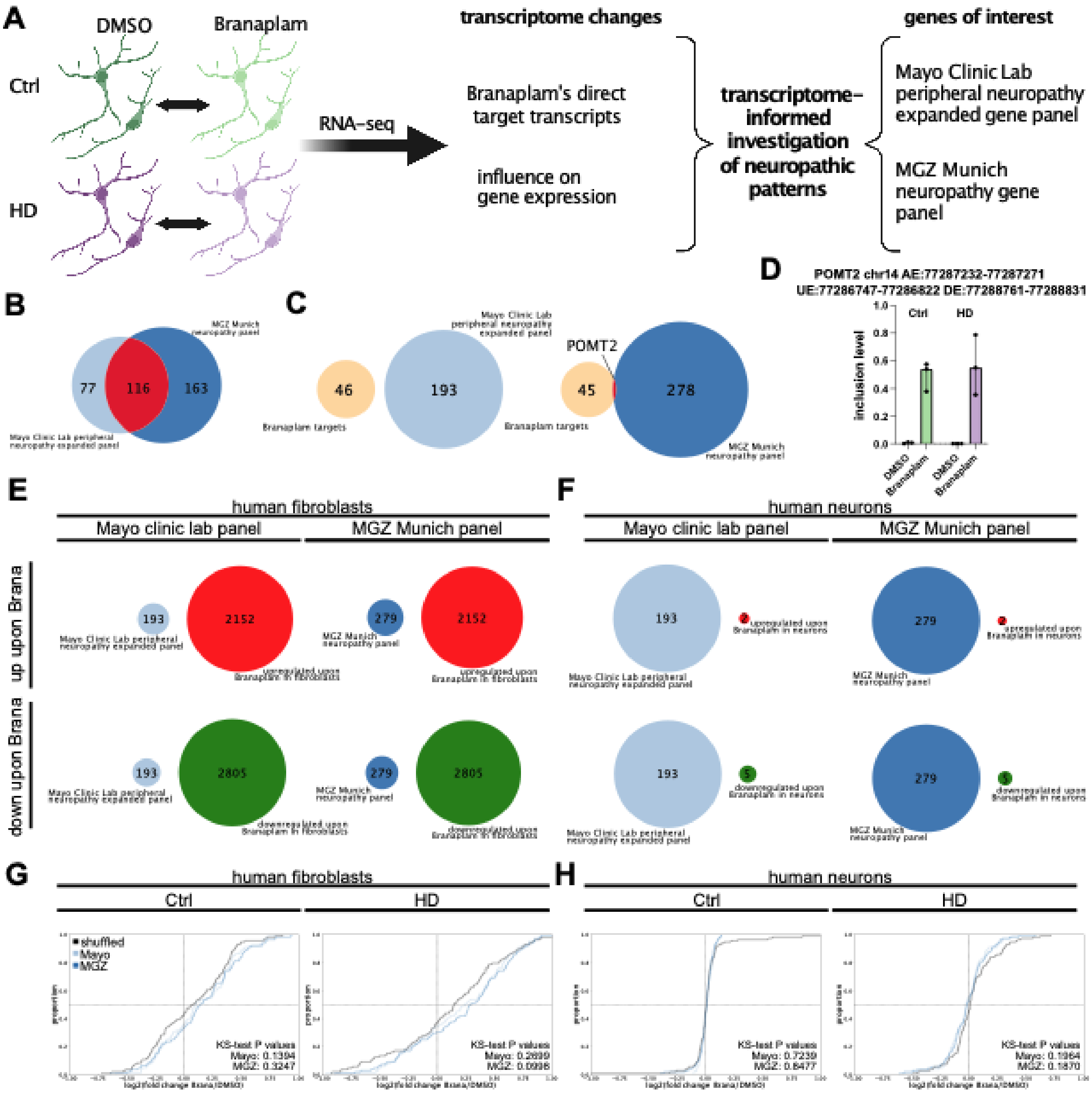

Figure S1, Related to Figure 1: Branaplam does not directly target hereditary neuropathy associated genes.

(A) Paradigm of analysis strategy. Published branaplam target genes and fibroblast and iPSC-neuron RNA-seq data [3] from Ctrl (greens) and HD (purples) with DMSO or 72 h 10 nM branaplam treatment (opaques) was used and compared to two genes in two diagnostic panels for hereditary neuropathies.

(B) Venn diagram showing overlap of genes (red) of the two neuropathy gene panels (light blue: Mayo Clinic Lab; blue: MGZ Munich).

(C) Venn diagram showing overlap (red) of previously determined branaplam targets (orange)[3] and gene list from Mayo Clinic Lab (light blue, left) or MGZ Munich (blue, right).

(D) Bar graph with inclusion levels of branaplam-targeting POMT2 exon in iPSC-neurons of Ctrl (greens) and HD (purples) with DMSO or branaplam (opaques) treatment. Top depicts exact location of event in GRCh38. AE: alternative exon, UE: upstream exon, DE: downstream exon. Data shown as median and interquartile range.

(E) Venn Diagrams of overlap of differentially upregulated (red) or downregulated (green) genes (DESeq2 adjusted P value < 0.05) upon 72 h 10 nM branaplam treatment in fibroblasts with Mayo Clinic Lab (light blue) or MGZ Munich (blue) neuropathy gene panel list.
(F) Venn Diagrams of overlap of differentially upregulated (red) or downregulated (green) genes (DESeq2 adjusted P value < 0.05) upon 72 h 10 nM branplam treatment in iPSC-neurons with Mayo Clinic Lab (light blue) or MGZ Munich (blue) neuropathy gene panel list.
(G) Cumulative distribution plot of Ctrl (left) or HD (right) fibroblast $\log_2$(fold changes) of branaplam vs. DMSO of genes in the Mayo Clinic Lab (light blue) or MGZ Munich (blue) list and randomly shuffled background of equal size (black). Significance calculated with 2 sample Kolmogorov-Smirnov (KS) test compared to shuffled background.
(H) Cumulative distribution plot of Ctrl (left) or HD (right) iPSC-neurons $\log_2$(fold changes) of branaplam vs. DMSO of genes in the Mayo Clinic Lab (light blue) or MGZ Munich (blue) list and randomly shuffled background of equal size (black). Significance calculated with 2 sample Kolmogorov-Smirnov (KS) test compared to shuffled background.

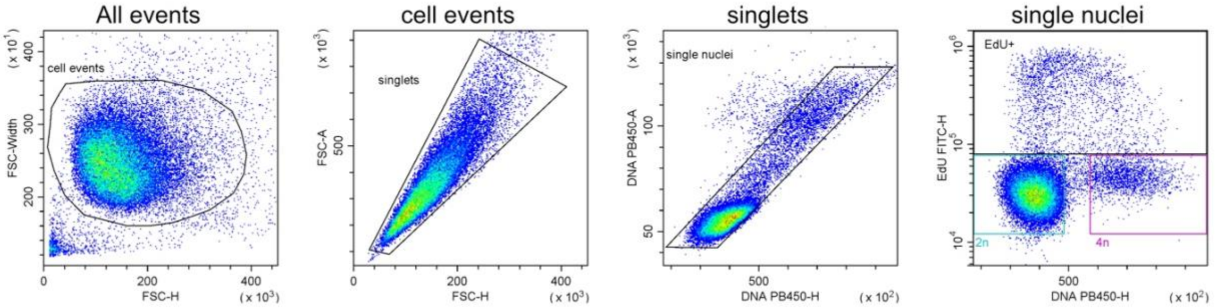

Figure S2, Related to Star Methods Cell cycle assay: FACS analyses for cell cycle assay
Gating strategy for cell cycle assay of human fibroblasts.

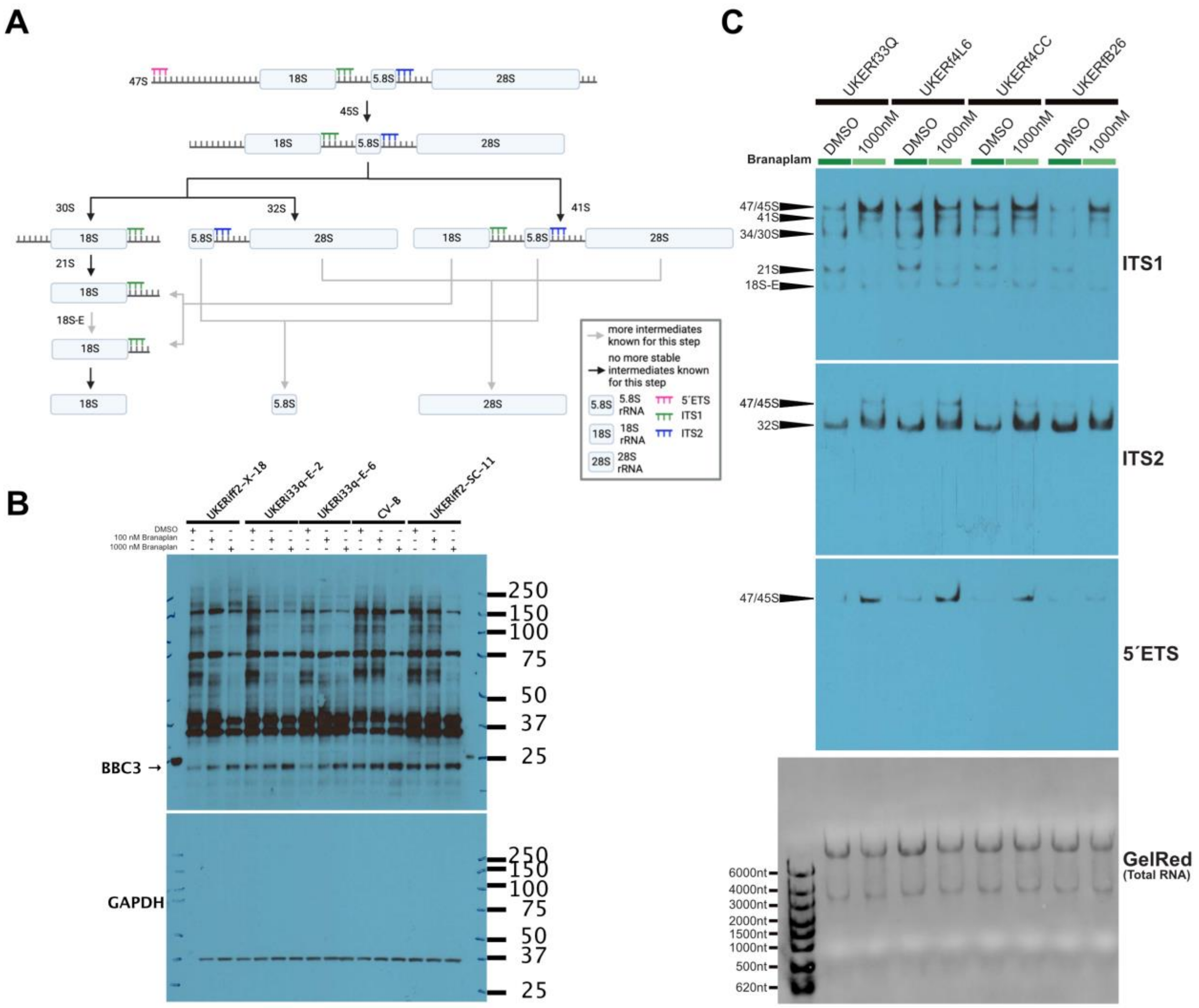

Figure S3, Related to Figure 3: uncropped BBC3 (PUMA) western blot and rRNA northern blot of fibroblasts treated with branaplam

(A) Northern blot analysis of 4 Ctrl fibroblast lines treated with DMSO or 1000 nM Branaplam using 3 different probes (ITS1, ITS2, 5'ETS) and GelRed staining of the membrane to visualize total RNA loading. Arrowheads on the right indicate rRNA intermediates.

(B) Paradigm of simplified rRNA metabolism pathway. Indicated in the pathway is the binding of the 3 probes used (ITS1, ITS2, 5'ETS) to detect rRNA intermediates.

(C) Uncropped western blot from main Figure 3D.
